# The Silent Period of Evidence Integration in Fast Decision Making

**DOI:** 10.1371/journal.pone.0046525

**Published:** 2013-01-21

**Authors:** Johannes Rüter, Henning Sprekeler, Wulfram Gerstner, Michael H. Herzog

**Affiliations:** 1 Laboratory of Psychophysics, Brain Mind Institute, École Polytechnique, Fédérale de Lausanne, Lausanne EPFL, Switzerland; 2 Laboratory of Computational Neuroscience, Brain Mind Institute and School of Computer and Communication Sciences, École Polytechnique Fédérale de Lausanne, Lausanne EPFL, Switzerland; University of Sydney, Australia

## Abstract

In a typical experiment on decision making, one out of two possible stimuli is displayed and observers decide which one was presented. Recently, Stanford and colleagues (2010) introduced a new variant of this classical *one-stimulus* presentation paradigm to investigate the speed of decision making. They found evidence for “perceptual decision making in less than 30 ms”. Here, we extended this one-stimulus compelled-response paradigm to a *two-stimulus* compelled-response paradigm in which a vernier was followed immediately by a second vernier with opposite offset direction. The two verniers and their offsets fuse. Only one vernier is perceived. When observers are asked to indicate the offset direction of the fused vernier, the offset of the second vernier dominates perception. Even for long vernier durations, the second vernier dominates decisions indicating that decision making can take substantial time. In accordance with previous studies, we suggest that our results are best explained with a two-stage model of decision making where a leaky evidence integration stage precedes a race-to-threshold process.

## Introduction

**Figure 1:**
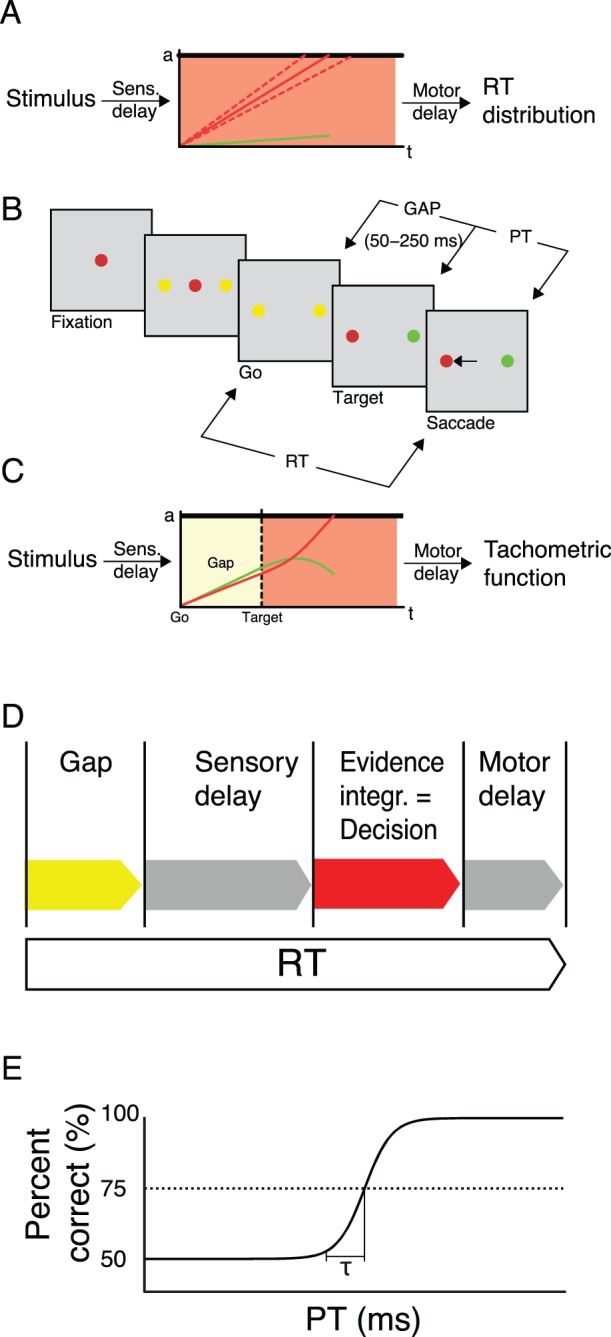
Rapid decision making and compelled responses. **A.** Race-to-threshold model. Evidence for each stimulus alternative is accumulated until a threshold *a* is crossed (solid red and green lines for either of the two stimulus alternatives, respectively). The evidence for the stimulus alternative that crosses the threshold first determines the decision (here, red). Reaction times vary because the accumulated evidence varies across trials due to noise (dashed lines). **B.** Compelled response paradigm [20]. A red or green central fixation dot is presented. Then, two peripheral yellow dots appear. The response period starts when the central fixation dot disappears (“GO”). After a variable “GAP” of 50–250 ms duration, one of the dots turns red and the other green. In order to receive reward, macaque monkeys had to execute a saccade to the dot which matched the color of the fixation dot within 600 ms after the GO signal. The difference between the reaction time (RT) and the gap duration (GAP) is the processing time (PT), i.e., the duration for which stimulus evidence is available for decision making. **C.** Accelerated race-to-threshold model according to Stanford *et al.* [20]. During the GAP period, noise drives the decision process for both stimulus alternatives. If gap durations are long, the noise drives evidence across the threshold leading to a “guess” decision (not shown). If gap durations are short, the evidence for the correct answer quickly increases (red line) rapidly leading to a decision, while the evidence for the other alternative decreases (green line). **D.** Total reaction times (RT) in the compelled-response paradigm consist of the gap duration, the constant sensory and motor delays and the actual decision making time which is identical to the time of evidence integration. In classical decision making experiments, no gap is presented. **E.** In the compelled- response paradigm, the decision making time can be derived from the tachometric function. The tachometric function plots performance (percent correct) as a function of processing time 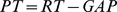, i.e., the duration stimulus evidence is available for decision making. The non-decisional time (sensory and motor delay) is thought to be reflected in the flat part of the tachometric function where performance is around 50%. The rise time 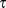 from chance level 50% performance to the 75% correct responses threshold (dashed line) is a measure for evidence accumulation. Stanford et al. (2010) estimated this time to be about 30 ms [20].

Humans can recognize objects and animals within a fraction of a second suggesting that decisions can be made very quickly [1]. In a typical decision making experiment, a stimulus is presented and observers have to decide as fast and accurately as possible between two response alternatives. The viewing time of the stimulus is unlimited [2], [3]. The speed of decision making is traditionally quantified in terms of reaction times, i.e., the time from stimulus onset to motor response onset. However, reaction times do not only include the decision making time but also sensory and motor delays, which makes it difficult to disentangle the three [4]. Decision making is often assumed for simplicity to be a one-stage process where stimulus evidence is accumulated until the evidence for one of the two response alternatives crosses a threshold [5]–[19] (Fig. 1A).

Recently, Stanford and colleagues (2010) introduced a novel compelled-response paradigm [20] (Fig. 1B). In this paradigm, monkeys were first shown a red or green fixation dot. Next, two yellow peripheral dots were displayed indicating the two potential target locations (“GO”). Then, the fixation dot disappeared and monkeys started a saccade to either target location. After a variable duration (“GAP”), one of the yellow dots turned red, the other green. Monkeys were required to saccade to the dot matching the color of the fixation dot within 600 ms after the disappearance of the fixation dot to receive reward (Fig. 1B). Saccades longer than 600 ms were not rewarded even when correct. Hence, viewing time of the stimulus was variable depending on gap duration. For long gap durations, saccades may be initiated even before the target is presented on the screen. Hence, no or little stimulus evidence is accumulated leading to chance performance because both alternatives are equally likely to cross the threshold first. The shorter the gap durations are the more stimulus evidence is available. Evidence for correct responses increases and evidence for incorrect responses decreases (Fig. 1C). Hence, the gap duration (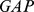) determines the processing time (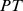) available for decision making, i.e., 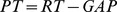, 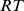 for reaction times (Fig. 1D). Stanford and colleagues plotted 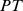 versus the percentage of correct responses (tachometric function; Fig. 1E; Stanford and colleagues (2010) plot the *effective* processing time 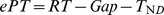, where 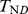 is the non-decisional time (sensory and motor delays) as estimated by the duration of the flat part of the tachometric function [20]). The flat part of the tachometric function at about 50% correct responses reflects the *constant* sensory and motor delays. The rise time 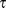 from 50% to 75% correct responses is a measure for the accumulation of evidence. Stanford et al. (2010) found 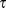 to be around 30 ms, suggesting that decision making is a very rapid process. Here, we extended the compelled-response paradigm to a two-stimulus presentation paradigm. Our results show that decision making can take substantial amounts of time.

## Results

**Figure 2:**
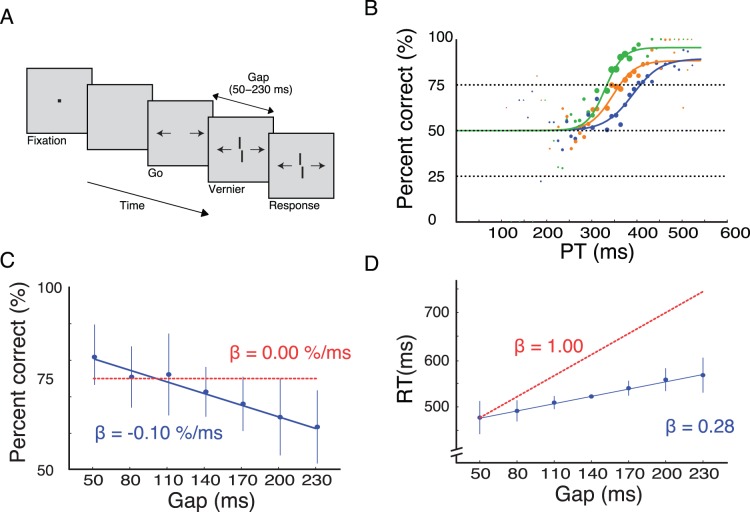
Compelled response paradigm for vernier stimuli [20]. **A.** Vernier only condition. Each trial started with the presentation of a fixation dot presented with a random duration of 800–1200 ms. After a blank period with a random duration of 200–400 ms, two arrows were presented (“GO”) starting the response period of 600 ms. After a variable gap of 50–230 ms, a vernier was presented until the end of the response period. Human observers indicated the offset direction as quickly as possible. Gap durations were presented randomly interleaved. **B.** Performance of three observers in the vernier only condition. We determined the time from the “GO” signal to the response and subtracted the gap duration in each trial (PT). Performance is at about chance level from 0 ms to about 250 ms. Then, performance quickly rises reaching 75% correct responses within 63 ms on average. The dot sizes reflect the number of responses per 10 ms bin. **C.** Performance as a function of gap duration. Had observers always waited for the targets, the curve would be a constant (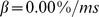; red dashed line). However, the longer the gap, the worse is performance (blue dots; fitted linear regression, blue line: 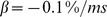). Error bars represent the standard deviation of the mean (SD) for three observers. **D.** Reaction times as a function of gap duration. Reaction times increase only moderately (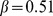) indicating that observers react immediately to the go signal (blue dots). The red curve (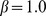) shows hypothetical RTs if observers had held the response until the cue appeared.

First, we adapted the compelled-response paradigm for human observers and vernier stimuli. A fixation dot appeared which was followed by two arrows (“GO”) indicating the two response alternatives (left/right push button presses). After a gap of a variable duration, a vernier was presented. The vernier consisted of two slightly offset vertical segments. The task of the observer was to report the position of the lower segment with respect to that of the upper segment as quickly as possible (Fig. 2A). As Stanford and colleagues, we computed the processing time 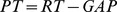 and determined percentage of correct responses for any given 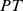 (tachometric function). We found that the tachometric function was flat for about 250–300 ms, followed by a rapid transition. 75% correct responses (Fig. 2B) were reached at 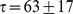 ms (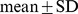) on average, a value well comparable to Stanford and colleagues (2010).

To properly determine the processing time, it is important that observers, indeed, start the response with the “GO” signal and do not wait until the target appears [21]. If observers were holding the response, PT would be independent of gap duration and hence mis-calculated. The percentage of correct responses decreased with increasing gap duration indicating that observers indeed started the response with the “GO” signal (psychometric curve; slope factor 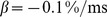, significantly different from 0.0; t(5) = 13.3160, p = 0.002; Fig. 2C). In addition, reaction times increased only moderately with gap duration as determined by linear regression (chronometric curve; slope factor 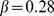, significantly different from 1.0; t(5) = 46.66, p = 0.004; Fig. 2D). Hence, for a single vernier, our version of the compelled-response paradigm leads to very similar results as found by Stanford et al. [20].

**Figure 3:**
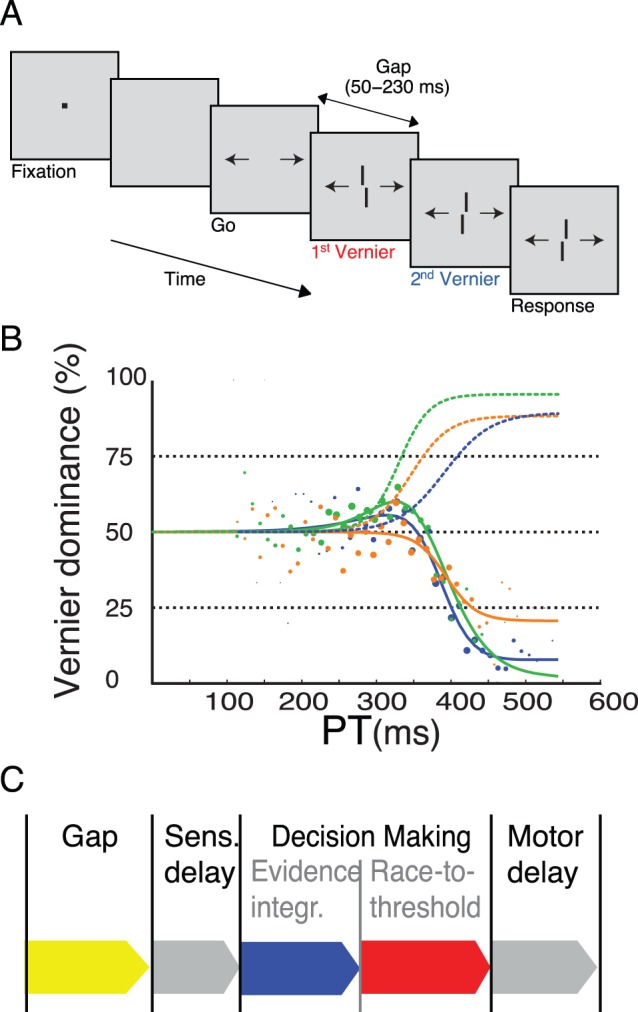
Compelled two-vernier stimulus response paradigm. **A.** The first vernier was presented for 80 ms followed by a second vernier with the opposite offset direction than the first vernier. The second vernier was presented until the end of the response period. **B.** For three observers, data are shown as percent dominance, i.e., the percentage of responses in accordance with the first vernier. Hence, when the first vernier dominates performance, dominance is above 50%; if the second vernier dominates dominance is below 50%. Dashed curves denote performance in the vernier only condition for the three observers (same as in Fig. 2B). The solid lines show performance in the two-vernier condition. The second vernier dominates strongly. **C.** A two stage model in which evidence is integrated first in a slow visual buffer and then fed into a fast race-to-threshold process. In one stimulus paradigms, the evidence integration is “invisible” because it is subsumed in the flat part of the tachometric function and, thus, confused with sensory and motor delays.

**Figure 4:**
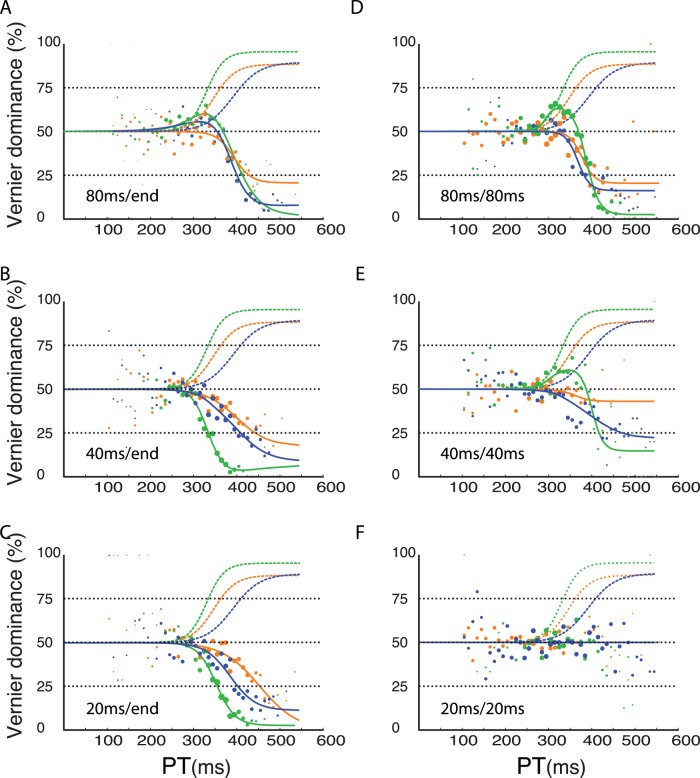
Results of compelled two-vernier stimulus response paradigm. The first vernier was presented either for 80 ms (A, D), 40 ms (B,E) or 20 ms (C, F). The second vernier was presented either until the end (“end”) of the response period (A, B, C) or for the same duration as the first vernier (D, E, F). Dashed curves are re-plotted from Fig. 2B and denote performance in the vernier only condition and are identical in all six plots. Solid lines indicate performance in the two-vernier conditions. When the second vernier was presented until the end of the response period, the second vernier clearly dominated performance (A–C; A is the same as Fig. 3B). Performance is very similar in all three conditions. When the second vernier was presented for the same duration as the first one, dominance of the second vernier decreased when durations of both verniers decreased (D–F). When the first and second vernier are presented for 20 ms each, dominance remains at about the 50% level for all subjects (F), i.e., both verniers cancel each other out. The tachometric function is flat (all three lines are on top of each other and only the blue line is visible). Except for one observer (green line), there is no evidence that evidence for the first vernier escapes integration with the second vernier. For this observer, it seems that in the 80/80 ms and 40/40 ms, the first vernier dominates for short gap durations.

In the second part of our study, we presented two verniers immediately one after the other. The first vernier was offset either to the left or right, whereas the second vernier was always offset in the opposite direction (Fig. 3A). Hence, if the first vernier was offset to the left, the second vernier was offset to the right and vice versa. In the first condition, we presented the first vernier for 80 ms and the second vernier until the end of the response period. If perceptual evidence were directly fed into a rapid decision process as assumed by most race-to-threshold models, the performance should be dominated by the first vernier, because the evidence for the first vernier would quickly cross threshold. In contrast to this prediction, we found that the second vernier strongly dominates the responses for all three observers (Fig. 3B). The dominance of the second vernier is largely unaffected by the duration of the first vernier indicating cancelation before the race to threshold process (Fig. 4A–C). In the second condition, both verniers were presented with the same duration. Still, the second vernier dominated except for the 20/20 ms condition where no dominance of either vernier was found (Fig. 4D–F). These findings suggest that decisions are based on an accumulation of evidence that outlasts the 63 ms that we found in the first part of the study. Therefore, the rise time of the tachometric function does not reflect the full decision process, but at least partly ignores a silent period of, as we will suggest, evidence integration.

## Discussion

In the first part of the experiment, we showed that decision making in vernier offset discrimination takes only a few tens of milliseconds. This finding is well in line with the findings of Stanford and colleagues who found evidence for “perceptual decision making in less than 30 ms” in monkeys [1], [21], [22]. The difference in duration between the two studies is most likely based on differences in task difficulty and faster reaction times in monkeys in general.

Stanford and colleagues (2010) fit their data with a one-stage race-to-threshold model where the stimulus directly drives evidence accumulation towards a threshold [3], [6]–[20], [23]. A decision is made when evidence accumulation crosses the threshold. Information that arrives after threshold crossing does not change the decision. We tested this prediction in the second part of the experiment by presenting two verniers with opposite offset directions (Fig. 3A). If decision making takes less than 63 ms, as estimated in the one vernier condition (Fig. 2B), the first vernier should dominate performance when presented for durations longer than 63 ms. Hence, performance in the condition, where both verniers are presented for 80 ms, should be very similar to the one vernier condition with 80 ms. The accumulated evidence for the first vernier should have crossed the threshold before the second vernier even entered the decision process. However, this was clearly not the case (Fig. 3B). The second vernier dominates in the two vernier condition and performance curves for the two conditions are rather mirror symmetric.

How can these results be explained? In a recent study, we investigated decision making with the one vernier fusion paradigm, i.e., a no compelled response paradigm. We found that accuracy and reactions times were not easily be explained with classical one-stage models [24]. For this reason, we proposed a two-stage model in which evidence integration and the race-to-threshold are not one and the same process in accordance with previous findings [25]–[27]. In our model, stimulus evidence is first integrated in a buffer after sensory transmission (Fig. 3C). Because of the buffering, evidence is not directly fed into the race-to-threshold process. This period is invisible in one-stimulus paradigms because subsumed in the flat part of the tachometric curve (which is usually attributed to the non-decisional time of sensory and motor delays only). The output of the buffer is, then, fed into a race-to-threshold process. The fast race-to-threshold process is reflected in the rapid transition from chance level performance to 75% correct responses [20]. Evidence integration in the sensory buffer must exceed 80 ms because the second vernier dominates even when the first vernier is presented for 80 ms. In our model, the buffer is implemented as a leaky integrator leading to “forgetting” of the evidence for the first vernier, thus, explaining the dominance of the second vernier. Performance curves are very similar when the first vernier is presented for various durations (Fig. 4A–E) suggesting that the second vernier, presented until the end of the 600 ms response period, fully “cancels” the first vernier during evidence integration, i.e., before the race-to-threshold process starts. This holds also true when the two verniers have equal duration, except for the 20/20 ms condition, where none of the two verniers dominates and performance remains at 50% dominance.

Our two stage model is well in accordance with previous work showing that sensory processing, e.g., motion processing or contrast detection, precede decision making [25]–[27]. This sensory processing stage is very comparable to our evidence integration stage for our high contrast vernier stimuli. A two-stage model might also explain the difference between the present study and the one by Stanford and colleagues (2010) which aimed to estimate the timing of the motor decision process. If “sensory” evidence integration precedes a motor decision process, then, our data indicate that indeed the motor decision process may well be very rapid. However, in addition to this fast motor decision process, a slow evidence integration stage adds to the entire decision making process.

These considerations rely on the assumption that decision making in two- and one-stimulus presentation paradigms proceed along the same lines. It might be that, because of the conflict between the two verniers presented in one trial, more complex mechanisms are in operation in two-stimulus presentation paradigms than if only one stimulus is presented. For example, it may be that there is strong inhibition between the vernier detectors in the two-vernier presentation paradigm but none if only one vernier is presented. However, whatever the mechanisms, our vernier fusion paradigm shows evidence for a silent integration process in decision making which is not visible in one-stimulus presentation paradigms. We like to mention that feature fusion is not restricted to verniers but occurs with all sorts of stimuli [28], [29].

When long stimulus durations exceed the timing of evidence integration, the difference between evidence integration and thresholding disappears [3], [4]. Therefore it is not surprising that evidence integration and thresholding are not distinguishable for longer stimulus durations [16], [17], [30], [31].

In summary, we have shown that reaction times can be substantially long in a vernier fusion paradigm. The compelled response paradigm, using single stimulus presentations and combined with a race to threshold model to estimate the non-sensory time, underestimates the *total* decision time in the vernier fusion paradigm. In accordance with previous work, we suggest that decision making is a two-stage process where evidence integration precedes a race-to-threshold process. In *one-stimulus presentation paradigms*, this evidence integration cannot be disentangled from sensory and motor delays because they are subsumed in the flat part of the tachistosopic function and therefore their timing cannot be determined. The timing of evidence integration can only be determined with *two-stimulus presentation paradigms*, where the two stimulus alternatives are presented in each single trial. We found that the *entire* decision making process clearly exceeds 80 ms.

## Materials and Methods

### Ethics Statement

All participants signed informed written consent. The study was approved by the Commission cantonale (VD) d’éthique de la recherche sur l’être humain (Lausanne, Switzerland) and conducted according to the principles expressed in the Declaration of Helsinki.

### Observers

Three human observers (1 female, aged 25–30 years) participated in the study. Participants had normal or corrected-to-normal visual acuity as measured by the Freiburg visual acuity test [32]. All observers were nave to the purpose of the study. Observers were paid students from the EFPL.

### Setup

Stimuli were presented on a Tektronix 608 X-Y display or a HP 1332A X-Y display. Both X-Y displays were equipped with a P11 phosphor and controlled by a PC via a fast 16-bit DA converter. Stimuli were presented at 80 cd/m^2^, a 1 MHz dot rate, a 200 Hz refresh rate, and a dot pitch of 200 µm. Viewing distance was 2 m. The room was dimly illuminated by a background light (0.5 lx) to prevent adaptation to scotopic vision. Stimulus contrast was close to 1.0.

### Stimuli and Task

We tested two different stimulus conditions. The first condition serves as a control and follows the compelled-response paradigm that was recently introduced by Stanford et al. [20]. Each trial started with a fixation dot (Fig. 2A). The disappearance of the fixation dot indicated the beginning of a trial. Then, two arrows appeared as a response prompt (“GO”). After a gap of a variable duration of 50–250 ms duration, in which observers had to prepare the response, a vernier was presented at the location of the fixation dot for the remainder of the response period. The vernier was composed of two slightly offset vertical segments (segments were 10′ (arc min) long, 0.5′ wide, separated by a vertical gap of 1′). The offset direction (left or right) was chosen randomly in each trial. Observers were asked to report the position of the lower segment with respect to that of the upper segment as quickly as possible and within the response period of 600 ms by pressing one out of two push buttons. Observers were instructed not to wait for the stimulus, but to initiate a response as soon as the GO signal was given. Observers received visual feedback if they did not respond within the response period. The gap duration was varied in 7 steps of 30 ms between 50 and 230 ms. All gap durations were presented randomly interleaved. A total of 400 trials were presented for each gap duration.

The second condition was identical to the control condition, except that a sequence of two vernier stimuli with opposite offset directions was presented in rapid succession, i.e. the interstimulus interval is 0 ms, instead of the single vernier (Fig. 3A). The offset direction of the first vernier was chosen randomly in each trial. The second vernier had an offset direction opposite to that of the first vernier. Observers perceived only one fused vernier with a small offset and were not informed that a sequence of two vernier stimuli was presented [33], [34]. The first vernier was presented for either 20, 40 or 80 ms and the second vernier for either the same duration or until the end of the response period.

In the first and second condition, the horizontal offset of the vernier(s) was 40″, which was more than twice the size of the offset discrimination threshold for each observer, as determined using the adaptive PEST procedure [35]. Thresholds were determined for a single vernier stimulus of 20 ms duration prior to the experiment.

### Reaction Time Analysis

Reaction times (RT) were measured from the “Go” Signal to the response. Reaction times exceeding the response period of 600 ms were not included in the analysis (less than 3% of the trials). In these trials observers are likely to have waited for the stimulus before initiating the response.

### Performance Measure

After subtraction of the gap durations from the reaction times, responses were analyzed in time bins of 10 ms. For each bin, we computed the dominance which is defined as the proportion of responses that matched the offset direction of the first vernier. Thus, values above 50% indicate dominance of the first vernier; values below 50% indicate dominance of the second vernier. 50% vernier dominance is the points of subjective equality, i.e., on average the first and second vernier stimuli equally contribute to performance. In the control condition, this measure is equivalent to the percentage of correct responses. We plot processing time (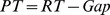) versus dominance.

### Fitting

The tachometric function was fit with one out of three parametric functions. The simplest function is given by the constant chance level 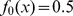. We fit the deviation of performance from chance level with either a single or a linear combination of two hyperbolic tangents:
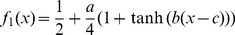
(1)

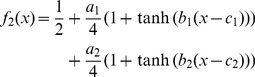
(2)


Which of the three functions is used for the fit was determined in a 10-fold cross-validation scheme: The parameters 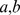 and *c* were fit to minimize the mean square error (MSE) on a training set consisting of 90% of the data. We then evaluated a test set consisting of the remaining 10%. This is repeated 10 times until all data have served as test data once. The test MSE is given by the sum of the MSEs on the 10 test sets. If a fit with two hyperbolic tangents improved the test MSE by more than 5%, we accepted the increase from 3 to 6 parameters. If the fit by a single hyperbolic tangent was not significantly better than the straight line, we plotted 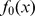. Once the function class is chosen, its parameters are fit using the full data set. The rise time 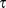 from chance level to 75% correct responses is defined by 25% divided by 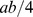, the slope of hyperbolic tangent at its inflection point.
